# DISABILITY: INDEPENDENT OR DEPENDENT VARIABLE IN GERONTOLOGICAL RESEARCH?

**DOI:** 10.1093/geroni/igae098.2174

**Published:** 2024-12-31

**Authors:** Michelle Putnam, Susan Stark

**Affiliations:** Simmons University, Boston, Massachusetts, United States; Washington University in St. Louis, St. Louis, Missouri, United States

## Abstract

A critical issue for bridging aging and disability research is developing a shared understanding of when disability should be employed as an independent or a dependent variable. To date, this discussion has rarely been had across researchers. Is disability a dependent variable, an outcome? Or does it contribute to outcomes as an independent variable? Or both? How disability is conceptualized, measured, analyzed, and interpreted matters for how we understand the role disability plays over the life course. Disability status is used as both an independent and a dependent variable in gerontological and disability/rehabilitation research. Research in both fields often focuses on reducing likelihood, duration, and severity of disability. A key distinction between fields is how each conceptualize disability – what it is and what it means – particularly given age of onset and type of disability. For example, in gerontological research, often positions disability as an individual trait that mediates the ability to live independently (typically reducing independence). However, disability/rehabilitation research commonly positions disability as a situational trait, or moderating variable associated with difficulty of living independently, but not causing inability to live independently. NIH’s recent inclusion of disability as a social determinant of health provides an opportunity for gerontologists to more deeply consider how disability is employed in research designs and why. We discuss how more consistent use of disability as an independent and/or dependent variable can help bridge aging and disability research and provide greater understanding of the role disability plays over the life course.

